# Predicting Risk of Postoperative Pneumonia by a Nomogram in Elderly Patients After Hip Fracture Surgery

**DOI:** 10.14740/jocmr6514

**Published:** 2026-07-31

**Authors:** Toshihiro Higashikawa, Kenji Shigemoto, Shintarou Iwai, Haruki Takigawa, Masahiro Hangyou, Tsugiyasu Kanda, Takeshi Horii, Masashi Okuro, Takeshi Sawaguchi

**Affiliations:** a Department of Geriatric Medicine, Kanazawa Medical University Himi Municipal Hospital, Himi, Toyama 935-8531, Japan; b Department of Geriatric Medicine, Kanazawa Medical University, Uchinada, Kahoku-gun, Ishikawa 920-0293, Japan; c Department of Orthopedics and Joint Reconstructive Surgery, Toyama Municipal Hospital, Hokubumachiimaizumi, Toyama, Toyama 939-8511, Japan; d Toyama Municipal Hospital, Hokubumachi, Imaizumi, Toyama 939-8511, Japan; e Kanazawa Medical University, Uchinada, Kahoku-gun, Ishikawa 920-0293, Japan

**Keywords:** Nomogram, Postoperative pneumonia, Hip fractures, Diuretics

## Abstract

**Background:**

This study aimed to develop a nomogram, a visual prediction model to predict the risk of postoperative pneumonia (POP) in elderly patients after hip fracture surgery.

**Methods:**

A retrospective analysis was conducted on 711 patients aged 65 and over who underwent surgery for hip fractures. A logistic regression analysis with a nomogram was used to identify independent risk factors for POP.

**Results:**

Out of the studied patients, 26 (3.7%) developed POP after surgery. Independent risk factors for POP included age, gender, body mass index (BMI), serum albumin levels, change in inflow E and mitral e’ annular velocities (E/e’), severity of fracture (fractured) and administration of diuretics. The results of both univariate and multivariate logistic regression analyses revealed that age, gender, serum albumin levels and diuretics showed significant increase in odds ratio, whereas no significant differences were found in BMI, E/e’ and fractured in odds ratios in POP patients. The area under the curve (AUC) of the nomogram model was 0.838, indicating discriminative ability.

**Conclusions:**

The nomogram developed in this study predicted the risk of POP in elderly patients with hip fractures. Using this model in clinical practice could allow for appropriate preventive measures to be taken for high-risk patients, potentially reducing the incidence of POP and improving patient outcomes.

## Introduction

Osteoporotic hip fractures are a significant health issue in the elderly, leading to functional decline, institutionalization, and even death [1]. The worldwide incidence of hip fractures among the elderly has been increasing, rising from 1.7 million cases in 1990 to an estimated 6.3 million by 2050 [2]. Approximately 10% of patients die within 1 year of a hip fracture in Japan [3], many due to postoperative complications such as wound infection, pneumonia, deep vein thrombosis, urinary tract infections, and cardiovascular and cerebrovascular events [4–8]. Among these complications, pneumonia, specifically postoperative pneumonia (POP) is one of the most crucial complications. In our internal survey, we found that the patients who started eating without swallowing problems after admission to the hospital were often complicated by aspiration pneumonia. This is presumably one of the reasons that patients developed POP. The repercussions of hip fractures in older individuals extend to a decline in the ability to carry out tasks necessary for independent living [9, 10]. In this patient group, instances of complicated pneumonia are commonly observed, yet reports that investigate the complications associated with pneumonia are scarce [10, 11]. Risk factors for POP include hypoalbuminemia, poor swallowing function, and heart failure [12]. What is more concerning is the escalating resistance to antimicrobial drugs, which complicates the process of intervention and treatment [6]. Hyperglycemia and a reduced platelet count are also recognized as factors contributing to POP following hip surgery [13]. Heart failure is classified into systolic and diastolic disorders, and ejection fraction is used as an indicator of systolic dysfunction and change in inflow E and mitral e’ annular velocities (E/e’) as an indicator of diastolic dysfunction [14–16].

Although the incidence of pneumonia after hip fracture and surgery is increasing, the relationships between hip fracture and pneumonia, as well as between hip fracture and eating and swallowing disorders, are still unclear. Although the mechanism is still unclear, certain background factors such as a high prevalence of underlying medical conditions may cause the progression of the frailty cycle, resulting in hip fractures, feeding and swallowing difficulties as well as cardiovascular disorders. The present retrospective study was conducted to identify the risk factors for POP and to create a nomogram model for predicting its occurrence, in order to determine strategies to improve the patient’s quality of life.

## Materials and Methods

This retrospective study included elderly patients admitted to the hospital from 2014 to 2020. Patients with preoperative E/e’ measured on the septal side by preoperative evaluation echocardiography were included. When the image quality of the echocardiogram was extremely poor due to conditions such as emphysema, severe obesity, or chest wall deformities, patients were excluded because accurate measurements were difficult to obtain. E/e’ and chest computed tomography (CT) were measured within 1 week, mostly 1 or 2 days before surgery, by cardiologist in the Himi Municipal Hospital. Demographic information was gathered by a team comprising physicians, specialist nurses, pharmacists, and medical staff. Additionally, this team assessed the presence of POP within the same department. POP diagnosis involved detecting dorsal inferior pulmonary infiltrates on postoperative chest CT scans. Cases were categorized as either POP positive or POP negative based on the presence of these infiltrates. The definition of POP followed the guidelines set by the Japanese Respiratory Society for managing hospital-acquired pneumonia [17], which includes criteria such as overt or suspected aspiration and signs of abnormal swallowing function or dysphagia, along with considerations of surgical and anesthetic factors. Electrocardiography measurements were performed in accordance with the recommendations of the Japanese Society of Echocardiography for routine clinical and research use [18]. Data collection was conducted through the review of electronic medical records at the institution.

### Statistical analysis

Continuous variables were presented as mean ± standard deviation, while categorical variables were shown as percentages.

Group comparisons were conducted using the Chi-square test and the Student’s *t*-test. Factors were compared between normal group and POP group. Based on the results of logistic regression, a nomogram prediction model for POP was developed. The nomogram is a figure or graph used to simplify the calculation or transformation of a certain function. In the case of nomogram using results from the logistic regression model, nomograms can be created as a simplified tool for obtaining risk of POP probability. First, a line is drawn vertically from the possible values of each variable, and the number of points on the “score” number line is calculated. The sum of these scores can then be plotted on the “total score” number line, from which a vertical line can be drawn on the “predicted risk of POP probability” number line to obtain an approximation of the probability. Regarding gender, 0 is male and 1 is female. E/e’ is a ratio, not a score, and has no unit. Regarding diuretics, the score 0–3 means the number of furosemide tablet (20 mg) administered per day. Fracture type was scored according to its severity, where a score of 0 indicated no fracture, 1 a neck fracture, 2 a trochanteric fracture, and 3 a neck + trochanteric fracture. The scores above 400 correspond to the risk of POP probability very close to 1 but not 1 (between 0.99 to 1.00).

For example, for 1 90-year-old male patient whose albumin level was 3.0, with E/e’ = 5, diuretic = negative, body mass index (BMI) = 30, fractured = neck, scores were estimated as follows: male 0 = 47, age 90 = 70, albumin 2.0 = 60, E/e’ 20 = 33, diuretics 0 = 90, BMI 30 = 21, fractured 1 = 6, indicated by thin vertical lines. The total score was 327 (47 + 70 + 60 + 33 + 90 + 21 + 6), which corresponds to about 0.9 of the risk of POP.

The nomogram was created using rms package in R, which includes the nomogram function to generate nomograms from a logistic regression model. Using data from the present study (n = 711), age, gender, albumin, E/e’, diuretics, and fracture status were included as independent variables, whereas POP status (positive/negative) was used as the dependent variable. The difference in binary outcomes was typically assessed by the area under the curve (AUC) of the receiver operating characteristic (ROC) curve. A prediction model was deemed acceptable with an AUC between 0.5 and 0.75, and an AUC greater than 0.75 indicated excellent discrimination. Additionally, decision curve analysis was performed to evaluate the net benefit of the nomogram in the decision-making process.

Net benefit was calculated as (true positives – false positives × weighting)/n, where n represented the total number of patients.

All statistical analyses were two-tailed, and statistical significance was set at P < 0.05. When the sample sizes for the two groups were N_1_ = 685 and N_2_ = 26, with a significance level of 0.05 and an effect size of 0.8, the power of the test was approximately 81%. Based on previous research, sufficient power was expected to be achieved. Data were analyzed using the freely available EZR (Easy R) software (Saitama Medical Center, Jichi Medical University, Saitama, Japan) [19].

### Ethical considerations

This retrospective study was conducted in accordance with the guidelines of the Declaration of Helsinki and received approval from the Clinical Research Ethics Committee of Hospital (Approval No.: 2018-06).

## Results

**Figure 1:**
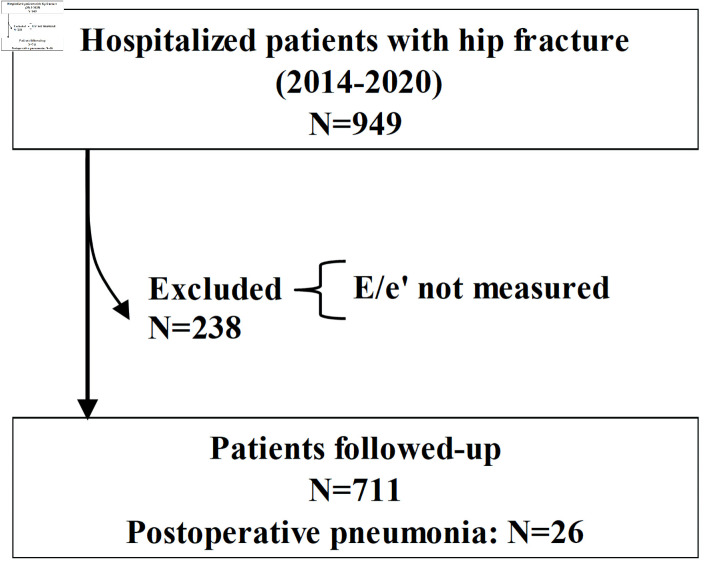
Flow chart for the study. E/e’: change in inflow E and mitral e’ annular velocities.

Of the 711 patients included in the study (Fig. 1), 26 (3.7%) developed POP after hip fracture surgery.

Patients with POP were more likely to be older, male, had lower serum albumin levels, required assistance with activities of daily living, and had a history of hypertension and previous bone fracture (Table 1).

**Table 1:** Patient Characteristics at Baseline

Variable	POP (–)	POP (+)	P value^a^
	N = 685 (96.3%)	N = 26 (3.7%)	
Age (years), mean ± SD	85.0 ± 7.1	88.5 ± 5.2	0.015
Gender, female, n (%)	561 (81.9)	12 (46.2)	< 0.01
BMI (kg/m^2^), mean ± SD	21.2 ± 4.1	19.6 ± 3.2	0.051
Fractured, trochanter, n (%)	398 (58.1)	17 (65.4)	0.547
Waiting time (h), mean ± SD	48.9 ± 130.4	51.1 ± 59.1	0.934
Systolic BP (mm Hg), mean ± SD	151.9 ± 27.1	148.5 ± 20.9	0.524
Diastolic BP (mm Hg), mean ± SD	78.6 ± 16.6	74.8 ± 16.3	0.256
Albumin (mg/dL), mean ± SD	3.7 ± 0.5	3.3 ± 0.6	< 0.01
Urinary retention, n (%)	112 (16.4)	6 (23.1)	0.368
ADL, assistance required, n (%)	127 (18.5)	9 (34.6)	< 0.05
Diuretics, n (%)	97 (14.6)	2 (7.7)	0.293
History of hypertension, n (%)	400 (58.4)	10 (38.5)	< 0.05
History of diabetes mellitus, n (%)	137 (20.0)	4 (15.4)	0.562
History of circulatory disease, n (%)	212 (30.9)	9 (34.6)	0.692
History of respiratory disease, n (%)	86 (12.6)	5 (19.2)	0.317
History of renal disease, n (%)	89 (13.0)	3 (11.5)	0.828
History of bone fracture, n (%)	375 (54.7)	20 (76.9)	< 0.05

^a^*t*-test (age, BMI, albumin), Chi-square test (others). Percentage of the bone fracture was calculated by left/(left + right) × 100. Fractured included neck, trochanter, neck + trochanter and others. Percentage of the trochanter was calculated by trochanter/fractured total × 100. Waiting time was the interval from time of admission to the time of surgery. The urinary retention was defined as a state of difficulty in micturition under storage of 400 to 500 mL of urine in a bladder after removal of the catheter. The ADL scale was used to conventionally assess degree of independence in everyday life on each case classified by four groups (J, A, B, C) where the ratio of the number of assistants (J + A) were calculated. POP: postoperative pneumonia; ADL: activity of daily living; SD: standard deviation; BMI: body mass index; BP: blood pressure.

Analysis of cardiac function revealed that E/e’ was significantly higher in patients who developed POP (Table 2).

**Table 2:** Characteristics of Cardiac Function

Variable	POP (–), n = 685 (96.3%)	POP (+), n= 26 (3.7%)	P value^a^
EF (%), mean ± SD	67.7 ± 9.5	64.2 ± 15.6	0.074
E/A, mean ± SD	0.69 ± 0.28	0.66 ± 0.24	0.694
E/e’, mean ± SD	14.3 ± 5.7	16.7 ± 7.3	0.035
LAD (mm), mean ± SD	32.4 ± 6.7	31 ± 6.1	0.372
IVCmax (mm), mean ± SD	12.6 ± 3.4	12.8 ± 2.7	0.768

^a^*t*-test. EF: ejection fraction; E/A: early filling/atrial filling; E/e’: change in inflow E and mitral e’ annular velocities; LAD: left atrial dimension; IVCmax: maximal diameter of inferior vena cava; POP: postoperative pneumonia; SD: standard deviation.

The results of logistic regression analyses showed significant differences in age, gender, BMI, albumin, E/e’ and diuretics, whereas no difference was found in fractured in odds ratios in univariate and multivariate analyses (Table 3).

**Table 3:** Odds Ratios on Univariate and Multivariate Logistic Regression Analyses

	Coeff.	SE	Univariate analysis			Multivariate analysis		
			Odds ratio (95% CI)		P value	Odds ratio (95% CI)		P value
Age	0.13	0.041	1.132	(1.045–1.225)	0.002	1.132	(1.045–1.225)	< 0.01
Gender	–2.28	0.484	0.190	(0.086–0.420)	< 0.01	0.102	(0.040–0.263)	< 0.01
BMI	–0.12	0.062	0.896	(0.806–0.996)	0.042	0.888	(0.787–1.002)	0.053
Albumin	–0.97	0.418	0.343	(0.176–0.666)	< 0.01	0.381	(0.168–0.864)	0.021
E/e’	0.04	0.035	1.061	(1.004–1.121)	0.037	1.039	(0.969–1.114)	0.280
Fractured	0.21	0.437	1.295	(0.602–2.784)	0.509	1.232	(0.523–2.899)	0.633
Diuretics	–1.37	0.670	0.350	(0.097–1.268)	0.110	0.255	(0.069–0.946)	0.041

BMI: body mass index; E/e’: change in inflow E and mitral e’ annular velocities; Coeff.: coefficient; SE: standard error; CI: confidence interval.

**Figure 2:**
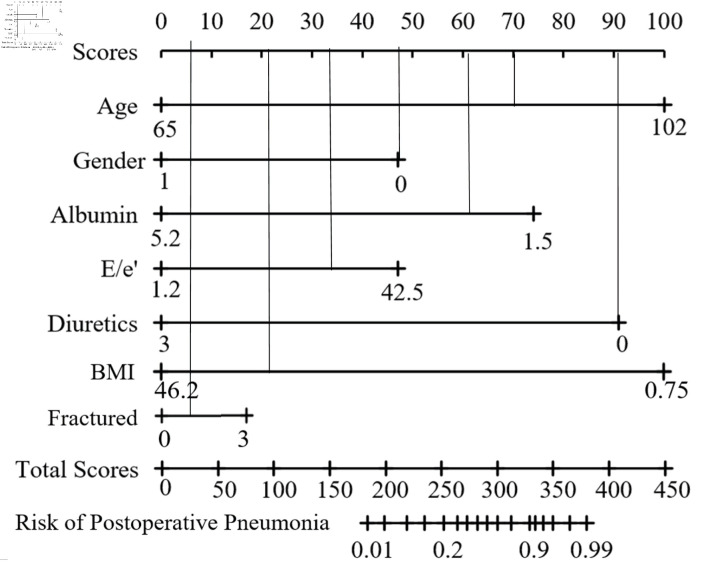
Predictive nomogram to assess the risk of postoperative pneumonia. BMI: body mass index; E/e’: change in inflow E and mitral e’ annular velocities.

The results of nomogram are summarized in Figure 2.

The total score 300 corresponds to 0.7 of the high-risk POP probability of the patient.

**Figure 3:**
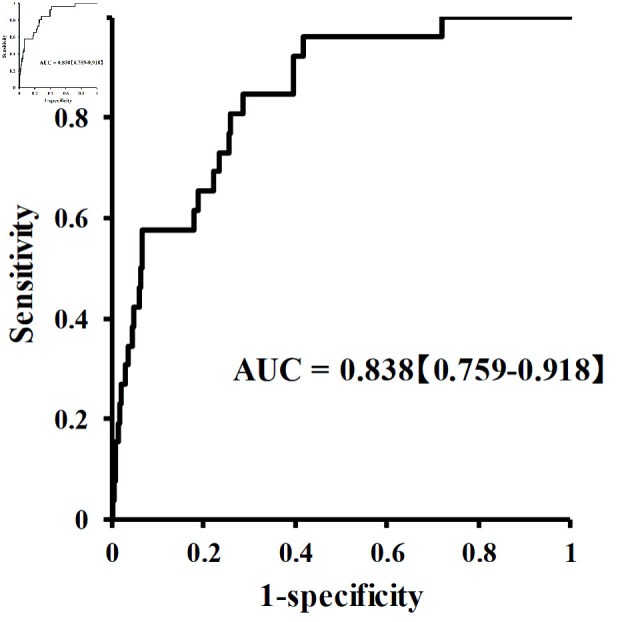
ROC curves of the logistic regression model. AUC: area under the curve; ROC: receiver operating characteristic.

The nomogram developed from this model demonstrated excellent discriminatory ability, with an AUC of ROC curve of 0.838 (0.759–0.918) (Fig. 3).

**Figure 4:**
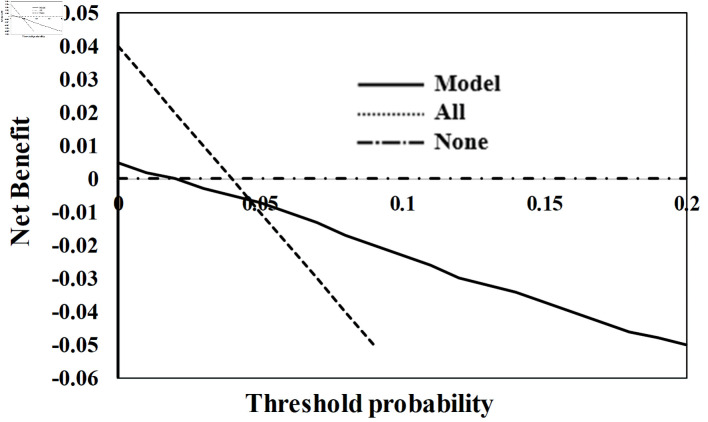
Decision plot of the nomogram for the probability of postoperative pneumonia.

This indicates that the nomogram can effectively distinguish between patients at high and low risk of POP. Decision curve analysis further supported the clinical utility of the nomogram, demonstrating a net benefit in decision-making within a threshold probability range from 0 to 0.02 (Fig. 4).

## Discussion

The present study has demonstrated that age, gender, serum albumin and use of diuretics are likely to be risk factors for POP in patients with hip fractures. In older patients, hip fracture is a frequent reason for hospital admissions. Roche et al demonstrated that 8.8% of elderly patients who had undergone hip fracture surgery were diagnosed with POP. This condition was identified as the most prevalent complication, leading to a higher mortality rate [8]. It has also been reported that 4.9% of patients with hip fracture who underwent surgery developed POP in China [20]. In this study, 3.7% of elderly patients with hip fractures were diagnosed with POP following their surgery, a figure marginally lower than those reported in earlier studies. This increase could be attributed to the change in phenomenon of aging in society in recent years. In the case of elderly patients with hip fractures, the risk factors contributing to the onset of POP are still subject to debate. Age, gender, BMI, hemoglobin levels, serum albumin, C-reactive protein (CRP), functional status, smoking habits, heart failure, chronic obstructive pulmonary disease (COPD), the interval before surgery, duration of the operation, blood transfusions, and the American Society of Anesthesiologists (ASA) classification might all potentially influence the development of POP [13, 21]. In this current research, age, gender, serum albumin levels, activity of daily living (ADL), E/e’, history of hypertension and bone fracture were recognized as independent predictors of POP (Table 1), whereas no significant variations were found in other variables. Earlier research indicated that complications following orthopedic surgery were often linked to higher BMI [22, 23]. However, malnutrition, a lower BMI, and decreased levels of serum albumin have consistently been identified as risk factors for POP [11, 24]. The rationale could be that elderly individuals frequently experience swallowing difficulties, potentially resulting in a lower BMI, malnutrition, and aspiration pneumonia. Furthermore, patients suffering from malnutrition often have compromised immune systems, making them more prone to infections. Numerous research papers and guidelines have advocated for elderly patients with hip fractures to receive surgical treatment at the earliest opportunity. The rationale is that prompt surgery can decrease the risk of postoperative complications and lower mortality rates [25–28]. Performing electrocardiogram on all patients leads to overscreening and may cause surgical delays. While the 90-day mortality rate is known, this study demonstrated the condition of cardiac function in elderly patients and the potential for cardiac function and pneumonia. B-type natriuretic peptide (BNP) has been known to be useful in selecting patients for echocardiograms, suggesting that it may help prevent overscreening [29]. As shown in Table 3, the use of diuretics and E/e’ were identified to be risk factors, and were therefore included in the multiple analysis. A case report indicated usefulness of the diuretics use for the treatment of pneumonia [30]. Several published studies have reported the development of nomograms for predicting the risk of POP in elderly patients after hip fracture surgery, identifying age, sex, albumin, time to surgery, intensive care unit (ICU), COPD, ASA > 2, etc., as risk factors [31–34]. The present study also found that age, sex and albumin were common risk factors, but diuretics was not common. Regarding prediction of these risk factors, nomogram depicted in Figure 2 is also a valuable tool for visualizing the output of predictive probabilities computed by a function (e.g., predictive or diagnostic model). Compared to the odds ratios, it is easier to understand by image visualization. The AUC of ROC 0.838 (0.759–0.918) indicates that the nomogram has excellent discriminatory ability for predicting POP probability after hip fracture surgery. In addition, the forecast of the decision plot showed that net benefit was in the quite narrow range (0–0.02) due to the large number of patients (n = 711). By calculating net benefit, we can quantitatively assess the balance of benefits and harms of using test results to predict and make treatment decisions.

In the future, it is considered necessary to incorporate the findings obtained from this study into clinical practice [35].

Although we did not retrospectively collect glycated hemoglobin (HbA1c) data from patients in our study, we excluded it based on previous studies indicating that elevated HbA1c levels are associated with a higher risk of anastomotic leaks and wound infections, but not pneumonia [36].

### Study limitations

The present study had multiple limitations. Firstly, being a retrospective study, it was subject to selection bias. Secondly, information regarding the use of drugs before and during surgery, as well as perioperative antibiotic use and care programs, was not gathered. Thirdly, the data presented in this article were derived from a single institution, which may limit generalizability. Fourthly, we made efforts to eliminate confounding factors, by including age and gender as covariates; however, other underlying confounding factors such as anesthesia type, duration of surgery and postoperative care cannot be neglected.

### Conclusions

In this study, we found that aging, gender, serum albumin level and diuretic use could be independent predictors of POP. Based on these factors, a nomogram and decision curve analysis (prediction model) were developed to predict the risk of developing pneumonia in elderly patients after hip fracture surgery. With this model, healthcare professionals can identify patients at high risk of pneumonia in advance and take more effective perioperative measures, which is expected to improve patient outcomes and reduce mortality.

## Data Availability

The data supporting the findings of this study are available from the corresponding author upon reasonable request.

## Abbreviations

POP: postoperative pneumonia
AUC: area under the curves
ROC: receiver operating characteristic
E/e’: change in inflow E and mitral e’ annular velocities
